# Analysis of Toxic Metals in Liquid from Electronic Cigarettes

**DOI:** 10.3390/ijerph16224450

**Published:** 2019-11-13

**Authors:** Naudia Gray, Mary Halstead, Nathalie Gonzalez-Jimenez, Liza Valentin-Blasini, Clifford Watson, R. Steven Pappas

**Affiliations:** 1 Centers for Disease Control and Prevention, Tobacco and Volatiles Branch, 4770 Buford Hwy, MS S110-3, Atlanta, GA 30341, USA; 2 Battelle Analytical Services, 2987 Clairmont Road, Suite 450, Atlanta, GA 30329, USA

**Keywords:** metals, inorganic, vaping, e-liquid, electronic cigarettes, ENDS, e-cigarettes, vapes

## Abstract

As the technology of electronic nicotine delivery systems (ENDS), including e-cigarettes, evolves, assessing metal concentrations in liquids among brands over time becomes challenging. A method for quantification of chromium, nickel, copper, zinc, cadmium, tin, and lead in ENDS liquids using triple quadrupole inductively coupled plasma mass spectrometry was developed. The method’s limits of detection (LODs) were 0.031, 0.032, 3.15, 1.27, 0.108, 0.099, 0.066 µg/g for Cr, Ni, Cu, Zn, Cd, Sn, and Pb respectively. Liquids analyzed were from different brands and flavors of refill bottles or single-use, rechargeable, and pod devices from different years. Scanning electron microscopy with energy dispersive spectroscopy further evaluated the device components’ compositions. Refill liquids before contacting a device were below lowest reportable levels (LRL) for all metals. Copper and zinc were elevated in liquids from devices containing brass. Cadmium was <LRL in all liquids and was not observed in device components. Cr, Ni, Cu, Zn, Sn, and Pb, reported in µg/g, ranged from <LRL to 0.396, 4.04, 903, 454, 0.898, and 13.5 respectively. Elevated metal concentrations in the liquid were also elevated in aerosol from the corresponding device. The data demonstrates the impact of device design and materials on toxic metals in ENDS liquid.

## 1. Introduction

ENDS (electronic nicotine delivery systems), particularly electronic cigarettes, have become a popular class of noncombustible tobacco products, especially among youth. From May 2017 to June 2018, e-cigarette sales increased by 97% [1]. In 2018, more middle and high school students used ENDS than any other tobacco product [2]. Exposure among youth and other users to toxic metals and other ENDS toxicants is not well studied but is of great importance. We previously published the results of aerosol analyses from various ENDS devices for the same seven toxic metals using a high purity fluoropolymer trap to avoid metals contamination [3]. With a few exceptions, low metal concentrations were found in the aerosol. The metals reported at the highest levels in aerosol were copper and zinc, apparently from brass components of the ENDS devices. In this study, many of the same device brands were examined for potential user metal exposures through dermal contact or aerosol inhalation.

Since ENDS’ introduction to the US, they have evolved through several design generations, with changes in the devices’ internal components and in the composition of the liquid, including the pH and form of nicotine [4]. The main categories of ENDS are cig-a-likes (single-use or cartridges with rechargeable batteries), tank/pen systems (refillable tanks with rechargeable batteries), mods (refillable and modifiable devices with rechargeable batteries), and pods (pre-filled pods with rechargeable batteries). For each type and brand, there are different inner component designs and materials which may contain the metals of interest for this study. Metals can be in contact with the liquid from the housing and vapor path (sometimes stainless steel), the heating element (often kanthal or nichrome), and the connections between the heating element and battery (brass connector, tin or tin-lead solder). The proximity of the metal components to the liquid can vary even when similar designs are used. The liquid itself is composed of humectants (propylene glycol and glycerol), with various levels of water, nicotine, pH modifiers, and flavors. In single-use or rechargeable cartridges, the liquid is often soaked into fibers and a heating element is wound around a wick. In pods, the liquid is in direct contact with the heating element within the pod. In tank or refillable systems, users add their desired liquid into a compartment. All of these various constructions make it difficult to generalize about the presence of metals in the liquid and aerosol.

Comparisons between liquids from ENDS refill bottles and liquids extracted from ENDS devices suggest that the components of ENDS devices are contaminating the liquid with metals [5,6]. One study showed varying metal concentrations among liquids from rechargeable cig-a-like devices and brands [7]. Identification of the internal metal components of ENDS devices suggests the composition of the parts includes chromium, nickel, copper, zinc, tin, and lead [8].

A validated method is described for quantitative analysis of toxic metals in ENDS liquids. Scanning electron microscopy with energy dispersive X-ray (SEM/EDS) also provided the elemental composition of specific components of select ENDS devices. The metal concentrations are reported in the liquids from new and older devices that had never been used for aerosolization to evaluate contact with different device components as a source of toxic metals. Information on constituent levels in the ENDS liquid solutions or suspension refills shows whether the constituents existed in the liquid before being added to the ENDS device. The toxic metal concentrations in ENDS liquid also correspond with levels of metals in the respective aerosols.

## 2. Materials and Methods

### 2.1. Sample Preparation for ENDS Liquid Analysis

All ENDS products are trademarks of their respective manufacturers and were obtained or ordered online from vendors in the greater Atlanta, GA, USA area. Products analyzed were from refill bottles (Joyetech, My Vapor Store), pods (JUUL), cartridges for rechargeable devices (Vuse, blu, 21st Century, Mistic), and single-use devices (NJOY, blu, Logic, Flavor Vapes). Liquid was sampled directly from containers for refillable devices or extracted by careful deconstruction of devices that had never been used for aerosolization. Analytical samples (100 to 200 mg) were diluted to 50 mL in previously acid cleaned class A polymethylpentene (PMP) volumetric flasks with 1% *v*/*v* double distilled hydrochloric acid (Ultrapur, Sigma, St Louis, MO, USA) and 1% *v*/*v* nitric acid (GFS, Powell, OH, USA, Environmental Grade, further purified by sub-boiling distillation in a perfluoroalkoxy (PFA) still, CEM, Matthews, NC, USA).

### 2.2. Analysis of ENDS Liquid Samples

Calibration standards were prepared by diluting NIST-traceable single element standards obtained from High Purity Standards (Charleston, SC, USA) into 1% *v*/*v* hydrochloric acid + 1% *v*/*v* nitric acid. The calibration blank consisted of the acid solution used to prepare the standards. The ENDS liquid calibration ranges for five standards were 0.100 to 20.0 µg/L chromium and nickel, 0.400 to 80.0 µg/L cadmium, tin, and lead, 8.00 to 1600 µg/L copper, and 4.00 to 800 µg/L zinc. When any sample concentrations were determined to be greater than the calibration ranges, they were diluted to fall within the calibration range. ENDS liquid concentrations in µg/L were multiplied by final analytical volume (0.0500 L) and divided by the mass of liquid to determine original undiluted concentrations in µg/g. Samples were analyzed in triplicate unless stated otherwise.

Two analytical quality control (QC) solutions were prepared with 200 µL of 50% propylene glycol (FCC grade, Sigma) /50% glycerol (Bioultra, Sigma) for matrix-approximation. Single element standards from a second source (Inorganic Ventures, Christiansburg, VA, USA) were diluted to 50.0 mL with 1% *v*/*v* hydrochloric acid and 1% *v*/*v* nitric acid, as for samples. Duplicate low and high QCs were analyzed before and after samples during each analytical run. Quality control was maintained using a modified Westgard plot [9] of the QC data using SAS software (Cary, NC, USA). When a QC failed, the run was either repeated, if sufficient ENDS liquid remained, or not reported, if insufficient inventory remained.

### 2.3. Validation

Accuracy was evaluated by quantifying low, mid, and high levels of all analytes spiked across the calibration range along with 200 µL of 50% propylene glycol/50% glycerol diluted to 50 mL in 1% *v*/*v* hydrochloric acid and 1% *v*/*v* nitric acid. Spiking solutions were prepared from a second source standard other than what was used to prepare the calibration standards used for quantitation. Accuracy ranged from 95–102%.

Calibration curve linearity was confirmed by residuals analysis of the linear regression of seven calibration curves with a coefficient of determination, R^2^, of ≥0.98. The calibration linearity was considered acceptable if R^2^ ≥ 0.99 for individual calibration curves using standard linear regression. The accuracy, precision, and residuals at each concentration level of the calibration curve were calculated and acceptable.

Matrix effects were assessed by comparing the slopes of analytes from a 10 calibrator standard curve spiked in 1% *v*/*v* hydrochloric acid and 1% *v*/*v* nitric acid to analytes spiked in 0.4% of 50% propylene glycol/50% glycerol (equivalent to 200 µL in 50 mL) in 1% *v*/*v* hydrochloric acid and 1% *v*/*v* nitric acid (*n* = 3). The matrix curves were prepared from a second source standard other than what was used to prepare the solvent blank curves. The percent errors of the matrix slope compared to the solvent blank curve were 2.19% or less for all analytes.

Method precision (evaluated as repeatability and intermediate precision) was assessed from 20 analytical runs of new preparations of duplicate low and high QCs over 20 days. Repeatability was calculated as within-run variation of duplicates, while intermediate precision as within-run and among-run variation. Table 1 presents the precision data. Repeatability was below 4.20% with the majority of analytes below 2.00%. Intermediate precision was below 2.50% for all analytes.

### 2.4. Instrument Parameters

Diluted ENDS liquid samples were introduced into an Agilent (Tokyo, Japan) 8800 QQQ-ICP-MS with an Elemental Scientific (Omaha, NE, USA) SC4-DX FAST autosampler via 0.64 mm peristaltic pump tubing with pump speed of 0.45 rps. Samples were further diluted by half by teeing in tubing of the same diameter with internal standard solution (20 µg/L rhodium and 2 µg/L iridium in 1% *v*/*v* nitric acid + 2% 2-propanol). Diluted samples were introduced into the plasma using a standard Peltier cooled PFA double pass spray chamber and C400 concentric PFA nebulizer (Savillex, Minnetonka, MN, USA). Plasma was maintained at 1550 watts RF power, 15 L/min plasma gas, 0.90 L/min auxiliary gas, optimized near 5.7 mm sampling position for low oxides. Nebulizer gas was optimized as needed for highest signal while maintaining cerium oxide formation below 2%. Lens parameters were optimized as needed with the exception of method and mode-specific parameters in Table 2.

### 2.5. Method Limits of Detection and Quantitation

Method limits of detection were determined according to Taylor’s prescribed method [10], with standard deviations of the five calibration standards and two QCs after 25 analytical runs plotted against concentrations with regression lines extrapolated to S_0_. S_0_ was multiplied by 3 to determine the methodological limit of detection (LOD). The final method LODs were experimentally confirmed.

### 2.6. SEM-EDS

**Figure 1:**
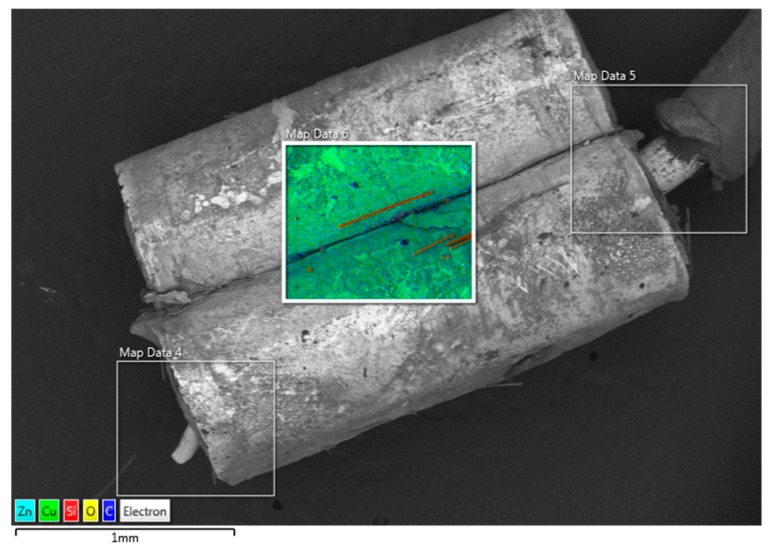
SEM image with EDS elemental mapping of a representative brass connector. Silicon- and oxygen-containing fibers are from the device wick.

Scanning electron microscopy was performed using an FEI Quanta 250 field emission instrument (Hillsboro, OR, USA) with Oxford energy dispersive X-ray (80 cm^2^) silicon drift detector (SEM-EDS). The SEM image in Figure 1 was obtained at 10.00 kV beam energy using an Everhart-Thornley detector in high vacuum mode at 6.7 × 10^−7^ Torr.

## 3. Results and Discussion

### ENDS Liquid Analysis

Lowest reportable level (LRL) was chosen from the higher of the LOD or the concentration of the lowest calibration standard (LSTD) expressed in terms of µg/g (Table 3).

Table 4, Table 5, Table 6 and Table 7 show results from all seven of our analytes for different e-liquid samples, noting the years the samples were received. Unless otherwise stated, the samples were analyzed within six months of receipt. Arsenic was consistently below the LRL in preliminary analyses of ENDS liquids during method development. Since there was no likely source for significant concentrations of arsenic in ENDS liquids, it was eliminated from further analytical consideration. Cadmium is not known to be a constituent of any component of ENDS devices. Although cadmium concentrations are relatively high in tobacco [11], cadmium was not found at detectable levels in ENDS liquids even when the liquids were obtained from older devices. To the best of our knowledge, no studies have found arsenic or cadmium in any ENDS device components.

For the detectable analytes, chromium and nickel were generally low for all brands. Heating elements made of nickel-chromium alloys have been proposed as sources of these metals, although these alloys are corrosion-resistant. Stainless steel, if used in the device construction, is an alternative source of these metals. Lead and tin were generally below LRL or at low levels. When lead was detected, tin was also at a reportable level, except for one product with detectable lead and <LRL tin. Internal solder joints are likely sources of lead or tin from devices in which these metals were measurable. Copper and zinc were detected in ENDS liquids obtained from samples with brass electrical connectors. Figure 1 shows a scanning electron microscope image of a representative brass connector using energy dispersive X-ray detector with elemental mapping. Copper and zinc are shown as the major metal constituents, displayed in light blue and green. Carbon is seen as dark blue dots, likely e-liquid residue, and the red fibers are silicates, consistent with the wick material used in disposable e-cigarettes.

In other studies, SEM/EDS confirmed the presence of chromium, nickel, copper, zinc, tin, and lead in the dissected cartomizers of devices [8]. There is limited published research on liquid from disposable cig-a-like or pod devices. Dunbar et al. reported lead levels ranging from 25.2–838.4 µg/L in liquids from 11 nicotine-free disposable devices [5]. Hess et al. analyzed liquid from 5 cartridge devices, reporting means ranging from 53.9–2,110 µg/L for chromium, 58.7–22,600 µg/L for nickel, 0.415–205 µg/L for cadmium, and 4.89–1,970 µg/L for lead, with much variation among results within the same brand [7]. In contrast to these previous studies, we report ENDS liquid metal concentrations in µg/g, rather than μg/L. There is no healthy level of inhalation exposure to toxic metals, but μg/L measurements may give the appearance of ENDS user exposure to extremely high average metal concentrations. Since the actual volume of aerosol transported in 10 puffs is often less than 100 μL, the highest concentrations would translate to the order of 1 μg per 10 puffs or less, if we assume 100% metal transport. Measurable cadmium has been reported in ENDS liquids [7] and in manuscripts on metals in aerosol [12,13]. However, there are no ENDS devices for which cadmium has been reported as a device component, nor did our SEM-EDS, liquid results, or previously reported aerosol results [3] determined detectable cadmium.

Polymer refill bottles of ENDS liquid were analyzed before contact with any ENDS device. The brands analyzed were Joyetech^TM^ Full Flavor Tobacco 18 mg nicotine (obtained in 2014 and two-years old when analyzed) and My Vapor Store^TM^ Gold Premium 24 mg nicotine (obtained and analyzed in 2016). All metal concentrations in these liquids were below the LRL. Studies have reported metal concentrations in refill bottles before and after the liquid contacted a device [6,13]. Another study analyzed the liquid from plastic refill bottles and from cig-a-like devices [5]. The studies showed that metals significantly increased after contact with a device and concluded that the devices transfer metals to the liquid. The My Vapor Store liquid was added to a Joyetech eGo tank system in our previous study, and the aerosol showed detectable nickel, copper, zinc, tin, and the highest of the analyzed lead [3].

Pod devices are of great interest because of their popularity among youth. Five flavors of JUUL were analyzed for this study. JUUL states online that pods have a stainless steel vapor path and nichrome heating element [14]. The liquid is visible and contained directly within the pod. This differs from the products in Table 5, Table 6, Table 7 and Table 8 which contain the liquid in a fiber sheet. The liquids in pods that were obtained for this study were lower in metal concentrations compared with other ENDS devices analyzed, although lot-to-lot variability cannot be ruled out. All analytes were below LRL, with the exception of nickel for some samples (Table 4). A previous study focusing on lead in ENDS liquid also found that the two JUUL flavors analyzed were below their calculated limit of quantitation [5]. Results in Table 4 were from pods analyzed within 3 months of receipt. The analysis of older devices may yield different results.

Deconstruction of the devices for visual and microscopic inspection revealed the likely sources of metals in the ENDS liquids. With the exception of a few devices, including NJOY and Vuse, the majority of the rechargeable and disposable devices analyzed had brass connections. The connection between the battery and the heating element of the Vuse device was a metal lead composed of iron, nickel, and chromium, according to SEM/EDS analysis. Two-year old and newly obtained Vuse Original and Menthol devices were analyzed. The NJOY King Menthol devices were analyzed within six months of receipt. The metal concentrations from these two brands are shown in Table 5. Copper and zinc are <LRL, as expected given the absence of brass. The tin in NJOY is likely due to the presence of a tin solder joint.

Two ENDS devices, blu and Logic, analyzed from different manufacturing years had major product design changes affecting the internal components. Metal concentrations from these devices are displayed in Table 6. The initial design was analyzed two years after acquisition and is compared with the new design, which was analyzed within six months of acquisition. All of the products contained brass connectors, resulting in elevated concentrations of copper and zinc. Age likely plays a role in the metal levels present in the liquid. The liquid in older products has prolonged contact with the metal components, potentially resulting in metal corrosion and leaching. However, corrosion and leaching cannot be ruled the absolute cause of the increased metal concentrations in the liquid in older products due to the design changes between the two product years.

Three 21st Century cartridge flavors were examined: Menthol, Regular, and Zero Nicotine (Table 7). Analysis was performed on products that were two years old and newer products of all three varieties. Observation under the light microscope showed that only the older Menthol and Regular flavored products contained brass connectors. This was reflected in elevated copper and zinc concentrations in liquid from these products. In this case, the differing concentrations between old and new products stems not from longer contact of the liquid or corroding device components, but from a design change that eliminated the brass connectors. This brand also had differences in product design within the same manufacturing year. The older Zero Nicotine product did not contain any brass connectors while older Menthol and Regular products did. Although the external design for all of these products was identical, the internal components and subsequent metal levels changed—an example of the rapid evolution of ENDS product design.

The major remaining question is whether elevated metals concentrations in ENDS liquids results in higher metals concentrations in the device aerosols. Table 8 compares the brands with the highest copper and zinc concentrations from our previously published data on metals in ENDS aerosols [3] with the liquid concentrations in the same brands. Unless otherwise noted, aerosol results were reported in triplicate, as previously described [3]. Aerosol was collected from an aerosol machine into a novel trap and rinsed with an acid solution. The brands with higher copper and zinc concentrations in the liquids also had higher copper and zinc concentrations in the aerosols from the respective devices. Many of the brands listed in Table 4, Table 5, Table 6, Table 7 and Table 8 correspond to aerosol data from our previous publication [3]. The analytes reportable in liquid were also detected in the aerosol generated by the same brands, excepting nickel, which was present in very low concentrations in JUUL liquid. In a few instances, analytes were reportable in the aerosol but were <LRL in the liquid of the same brand. This was mostly the case with copper, although copper levels for these samples were not elevated in the aerosol. The metals detectable in aerosol, but not in liquid, could have come from heating the device. While the transfer efficiency from liquid to aerosol is low for the relatively nonvolatile metals studied, knowledge of metal concentrations in ENDS liquids could help to predict product user exposure to metals at elevated levels from inhalation of the aerosol.

## 4. Conclusions

A method for the analysis of seven toxic metals in ENDS liquids was developed. Analyses of ENDS liquid that had not yet contacted metallic ENDS device components showed metal concentrations below the LRLs, confirming that detectable metals in liquids are derived from contact between the liquid and device components. Other studies have also concluded that the device itself contributes to metal contamination in the liquid [5,6,13]. The presence of brass connectors resulted in elevated levels of copper and zinc. Cadmium, a metal with relatively high concentration in tobacco, has not been found as a component used in ENDS devices. Its absence was reflected in below LRL cadmium concentrations for the liquid in all brands analyzed, as we previously observed in device aerosols. For the other analytes, concentrations ranged from <LRL to 0.396 µg/g for Cr, 4.04 µg/g for Ni, 903 µg/g for Cu, 454 µg/g for Zn, 0.898 µg/g for Sn, and 13.5 µg/g for Pb. Heating elements containing nichrome (an alloy of nickel and chromium) and stainless steel are found in some devices. Nickel and chromium are present in small amounts in some samples, although these metals were often below LRL. Corrosion from solder joints are likely the source of tin or lead found in a few liquids. These metals were generally below LRL or at low levels. JUUL pods have a unique design and were found to have all the metals analyzed below LRL or just above reportable levels for nickel.

The metals that were found in ENDS devices and liquids that are transported in aerosol can present various health risks to the user. The inhalation of toxic metals at any level is not safe for the user. However, it is not possible to generate a single factor to calculate transport efficiencies of metals from liquids into aerosols. The transport is influenced by many variables, including the constantly changing levels of corrosion within individual devices, differences in diameters and compositions of heating elements, the power provided to the heating elements that produce the heat for liquid vaporization, and the differences in mouthpiece design that affect nebulization. A high number of replicates of both the liquid and the aerosol per product would need to be analyzed in order to provide data that could be used to calculate ranges of potential exposure, keeping all other factors constant among specific products. Although some liquids presented here may be low in toxic metals, the various other constituents and properties of the ENDS liquid and aerosol can still present issues to the user. Analysis of other areas of concern, such as pH, nicotine, flavors, and volatile organic compounds, would provide a larger picture on the health consequences of ENDS.

Overall, data show that device components and construction play a large role in metal concentrations in ENDS liquids. Elevated levels of some metals in liquids from older devices included in our study may be due to different designs or extended contact between the liquid and metal components. In addition, constituent levels may change over time as substances in the liquid are oxidized and as internal device components in contact with the liquid degrade. The constant design changes in these devices make it difficult to predict metal concentrations in emerging products, even from the same brand. ENDS should continue to be monitored throughout varying years and lots as device and liquid changes can affect toxic metal levels. The metal concentrations in the liquids can be helpful in predicting inhalation exposures from the aerosols to ENDS users. Improvements to device designs could reduce exposure to these metals.

## Figures and Tables

**Table 1:** Method precision using 20 duplicate runs of quality controls (QCs)**.**

Precision (%RSD) (*n* = 20)				
Analyte	QC Sample	Mean	Repeatability (%)	Intermediate Precision (%)
**Cr**	Low Spike	2.02	3.70	2.03
	High Spike	15.1	2.58	2.04
**Ni**	Low Spike	2.03	4.16	2.34
	High Spike	14.7	1.47	1.16
**Cu**	Low Spike	204	1.33	0.814
	High Spike	1500	1.24	0.912
**Zn**	Low Spike	101	2.22	1.32
	High Spike	764	2.07	0.902
**Cd**	Low Spike	10.0	1.86	0.953
	High Spike	75	1.46	0.797
**Sn**	Low Spike	10.1	1.62	0.875
	High Spike	74.8	1.16	0.737
**Pb**	Low Spike	9.98	1.38	0.841
	High Spike	76.4	1.58	1.13

**Table 2:** ENDS Liquid Instrument Modes and Internal Standard Assignments.

Element Isotope	Instrument Mode	Cell Gas	Quantitated Ion	Quantitated Mass	Internal Standard
^52^Cr	MS-MS	NH_3_	^52^Cr(NH_3_)_2_^+^	86	^103^Rh(NH_3_)_4_^+^
^60^Ni	MS-MS	O_2_	^60^NiO^+^	76	^103^RhO^+^
^63^Cu	MS-MS	He	^63^Cu^+^	63	^103^Rh^+^
^68^Zn	MS-MS	He	^68^Zn^+^	68	^103^Rh^+^
^111^Cd	MS-MS	O_2_	^111^Cd^+^	111	^103^RhO^+^
^118^Sn	MS-MS	He	^118^Sn^+^	118	^103^Rh^+^
^206+207+208^Pb	MS-MS	He	^206,207,208^Pb^+^	206 + 207 + 208	^193^Ir^+^

Cell parameters for modes: 0.5 mL/min O_2_ cell gas with −20 V octopole bias, −8 V energy discrimination; 4 mL/min 10% NH_3_, 90% He cell gas with −18 V octopole bias, −8 V energy discrimination; 5 mL/min He cell gas with −18 V octopole bias, 3 V energy discrimination.

**Table 3:** Limits of detection and lowest calibration standard.

	Cr	Ni	Cu	Zn	Cd	Sn	Pb
**LOD (µg/g)**	0.031	0.032	3.15	1.27	0.108	0.099	0.066
**LSTD (µg/g)**	0.025	0.025	2.00	1.00	0.100	0.100	0.100

**Table 4:** Metal concentrations in electronic nicotine delivery systems (ENDS) liquids from JUUL pods obtained in 2018 (mean ± standard deviation, µg/g).

JUUL^®^	Cr	Ni	Cu	Zn	Cd	Sn	Pb
**Cool Mint**	<LRL	0.040 ± 0.009	<LRL	<LRL	<LRL	<LRL	<LRL
**Crème Brulee; Fruit Medley**	<LRL	<LRL	<LRL	<LRL	<LRL	<LRL	<LRL
**Mango**	<LRL	0.057 ± 0.008	<LRL	<LRL	<LRL	<LRL	<LRL
**Virginia Tobacco**	<LRL	0.091 ± 0.022	<LRL	<LRL	<LRL	<LRL	<LRL

**Table 5:** Metal concentrations from devices without brass connectors that were manufactured and analyzed in different years. Vuse devices are cartridges with rechargeable batteries, and NJOY devices are single-use (mean ± standard deviation, µg/g).

Brand	Cr	Ni	Cu	Zn	Cd	Sn	Pb
**Vuse Menthol** **2 years old** **2014**	0.396 ± 0.138	0.642 ± 0.078	<LRL	<LRL	<LRL	<LRL	<LRL
**Vuse Menthol** **2017**	0.300 ± 0.049	0.409 ± 0.080	<LRL	<LRL	<LRL	<LRL	<LRL
**Vuse Original** **2 years old** **2014**	0.243 ± 0.040	0.478 ± 0.045	<LRL	<LRL	<LRL	<LRL	<LRL
**Vuse Original 2017**	0.057 ± 0.027	0.387 ± 0.114	<LRL	<LRL	<LRL	<LRL	<LRL
**NJOY^®^ King Menthol 2016**	<LRL	0.148 ± 0.026	<LRL	<LRL	<LRL	0.355 ± 0.087	<LRL
**NJOY^®^ King Menthol 2017**	<LRL	0.188 ± 0.015	<LRL	<LRL	<LRL	0.119 ± 0.018	<LRL

**Table 6:** Metal concentrations comparing brands with design changes. The older devices were analyzed two years after acquisition (mean ± standard deviation, µg/g).

Brand	Cr	Ni	Cu	Zn	Cd	Sn	Pb
**blu^TM^ Classic Tobacco Black, High Nicotine Cartridge** **2 years old** **2014**	0.231 ± 0.018	4.04 ± 0.10	176 ± 6	3.00 ± 0.20	<LRL *	0.239 ± 0.010	<LRL
**blu^TM^ Classic Tobacco** **Single-use** **2017**	<LRL	0.050 ± 0.006	29.4 ± 3.2	9.32 ± 2.25	<LRL	<LRL	<LRL
**Logic Platinum** **2.4% Nicotine** **Single-use** **2 years old** **2014**	<LRL	2.59 ± 0.22	903 ± 27	454 ± 11	<LRL	0.898 ± 0.054	13.5 ± 0.4
**Logic Power** **2.4% Nicotine** **Single-use** **2017**	<LRL	0.731 ± 0.223	418 ± 58	140 ± 47	<LRL	0.216 ± 0.021	1.66 ± 0.93

* Duplicate results.

**Table 7:** Metal concentrations from 21st Century cartridges comparing older and newer samples (mean ± standard deviation, µg/g).

21st Century^®^	Cr	Ni	Cu	Zn	Cd	Sn	Pb
**Menthol Express** **2.0% Nicotine** **2 years old** **2014 ^§^**	<LRL	0.755 ± 0.318	319 ± 13	113 ± 2	<LRL	0.365 ± 0.142	1.35 ± 0.28
**Menthol Express** **2.0% Nicotine** **2016**	<LRL	0.363 ± 0.513	<LRL	<LRL	<LRL	<LRL	<LRL
**Regular Express** **2.0% Nicotine** **2 years old** **2014 ^§^**	<LRL	0.427 ± 0.058	205 ± 6	64.6 ± 1.9	<LRL	0.208 ± 0.164	<LRL
**Regular Express** **2.0% Nicotine** **2016**	0.033 ± 0.016	0.211 ± 0.068	<LRL	<LRL	<LRL	<LRL	<LRL
**Regular Express** **Zero Nicotine** **2 years old** **2014**	<LRL	0.452 ± 0.103	9.18 ± 0.73	12.5 ± 0.7	<LRL	<LRL	0.668 ± 0.015
**Regular Express** **Zero Nicotine** **2016**	<LRL	0.746 ± 0.342	<LRL	13.5 ± 0.3	<LRL	0.262 ± 0.210	0.691 ± 0.020

^§^ Brass connectors were observed in these cartridges.

**Table 8:** Mean ± standard deviation metal concentrations in ENDS brands from liquid (µg/g) and aerosol (ng/10 puffs).

Brand	Matrix	Units	Cu	Zn
**Mistic^®^ Traditional** **1.8% Nicotine** **Cartridge** **2017**	Liquid	µg/g	125 ± 12	30.2 ± 9.0
	Aerosol 1 year old	ng/10 puffs	488 *	265 ± 111
**Flavor Vapes^®^ Blueberry** **18 mg Nicotine** **Single-use** **2016**	Liquid 1 year old	µg/g	614 ± 64	339 ± 90
	Aerosol 2 years old	ng/10 puffs	251 ± 29	111 ± 9

* Duplicate results.
